# Novel Water Soluble Chitosan Derivatives with 1,2,3-Triazolium and Their Free Radical-Scavenging Activity

**DOI:** 10.3390/md16040107

**Published:** 2018-03-27

**Authors:** Qing Li, Xueqi Sun, Guodong Gu, Zhanyong Guo

**Affiliations:** 1 Key Laboratory of Coastal Biology and Bioresource Utilization, Yantai Institute of Coastal Zone Research, Chinese Academy of Sciences, Yantai 264003, Shangdong, China; qli@yic.ac.cn (Q.L.); cafeapril@163.com (X.S.); 2 Graduate School of Chinese Academy of Sciences, Beijing 100039, China; 3 Alliance Pharma, Inc., 17 Lee Boulevard Malvern, PA 19355, USA; guguodong011@gmail.com

**Keywords:** chitosan derivatives, 1,2,3-triazolium, free radical-scavenging activity

## Abstract

Chitosan is an abundant and renewable polysaccharide, which exhibits attractive bioactivities and natural properties. Improvement such as chemical modification of chitosan is often performed for its potential of providing high bioactivity and good water solubility. A new class of chitosan derivatives possessing 1,2,3-triazolium charged units by associating “click reaction” with efficient 1,2,3-triazole quaternization were designed and synthesized. Their free radical-scavenging activity against three free radicals was tested. The inhibitory property and water solubility of the synthesized chitosan derivatives exhibited a remarkable improvement over chitosan. It is hypothesized that triazole or triazolium groups enable the synthesized chitosan to possess obviously better radical-scavenging activity. Moreover, the scavenging activity against superoxide radical of chitosan derivatives with triazolium (IC_50_ < 0.01 mg mL^−1^) was more efficient than that of derivatives with triazole and Vitamin C. In the 1,1-diphenyl-2-picrylhydrazyl (DPPH) and hydroxyl radical-scavenging assay, the same pattern were observed, which should be related to the triazolium grafted at the periphery of molecular chains.

## 1. Introduction

Oxidation is an essential biological process to many organisms for the production of energy, and free radical at certain concentration is necessary for biological system. Free radicals in the human body can help transmit energy to sustain life motivation, kill bacteria and parasites, and help the body to eliminate toxins. However, the uncontrolled production of oxygen derived free radicals triggers many health problems such as Alzheimer disease, Parkinson’s disease, and ischemic-reperfusion injury [1]. Oxidative stress is caused by an imbalance between the production and consumption of oxidative species, and often involves reactions between free radicals and molecules of high biological importance, such as lipids, proteins, and DNA [2]. Moreover, restrictions over the use of synthetic antioxidants such as butyl hydroxyl anisd (BHA) and butylated hydroxytoluene (BHT) in food further strengthen the concept of using naturally occurring compounds as antioxidants [3]. Recently, many researches have reported the antioxidant capacity of nature polymers and saccharides, some of which have good free radicals scavenging ability and even have anticancer activity [4,5,6]. Polysaccharides derivatives are increasingly reported for their potential applications as antioxidants or free radical scavengers [7,8,9,10,11,12]. 

Chitosan is a natural, safe, and cheap polysaccharide produced from chitin, the major constituent of arthropods exoskeleton and fungi cell walls and the second renewable carbon source after lignocellulosic biomass. In addition to its low cost of production, chitosan also possesses several favorable biological properties such as biodegradability, biocompatibility and non-allergenicity. Chitosan itself has antioxidant activity on hydroxyl radicals with an IC_50_ of 0.48 mg/mL [13]. Many chitosan derivatives obtained by chemical modification were reported to have good antioxidant activity. Fan reported antioxidant activity of silk peptides grafted carboxymethyl chitosan, and the highest scavenging activity of 1,1-diphenyl-2-picrylhydrazyl (DPPH) was 24.86%, 91% of hydroxyl radical and 36.8% of H_2_O_2_ at the concentration of 0.5–2.5 mg/mL [14]. Double quaternized chitosan derivatives showed better scavenging ability than chitosan, with more than 90% scavenging indices against hydroxyl radicals and DPPH radicals at 1.6 mg/mL [15]. 

Triazole derivatives represent an interesting class of heterocyclic compounds; they possess many biological activities such as antimicrobial, anti-tubercular, anti-inflammatory and anticancer activities [16,17,18,19,20]. 1,2,3-Triazolium cations have recently been developed by quaternization of 1,2,3-triazoles with halogenide. Meanwhile, triazole and triazolium are expected to be more efficient anion captor [21], which may help stabilize the free radicals and have a good free radical scavenging ability. 

In this paper, we report the design and synthesis of a group of chitosan derivatives with 1,2,3-triazolium as substituent. Firstly, the C_2_-NH_2_ was modified as a quaternary ammonium salt. The quaternary ammonium salt was selected by virtue of water solubility, which could enlarge the application of chitosan as a food preservative or bioactive matrix. Afterwards, 6-azido-6-deoxy chitosan was synthesized as intermediate with azido group at C-6 of chitosan. Then, “click reaction” was selected as the key step to synthesize 1,4-disubstituted-1,2,3-triazolyl chitosan derivative. Terminal alkynes bearing benzene, pyridine, and thiophene were used in “click reaction” to introduce those groups at the periphery of chitosan chains. Afterward, the chitosan derivative bearing 1,2,3-triazolium was obtained by the alkylation of 1,2,3-triazolyl chitosan derivative. The chemical structures of the derivatives were characterized by Fourier Transform Infrared Spectroscopy (FT-IR) and ^1^H Nuclear Magnetic Resonance (^1^H NMR). We speculated that the aimed products might have improved antioxidant activity, and we investigated the free radical-scavenging activity of chitosan and the synthesized chitosan derivatives. Three types of classic free radicals, including hydroxyl radical, DPPH radical, and superoxide radical, were selected to estimate the radical scavenging ability of chitosan and the synthesized chitosan derivatives.

## 2. Results

### 2.1. Chemical Synthesis and Characterization

**Scheme 1:**
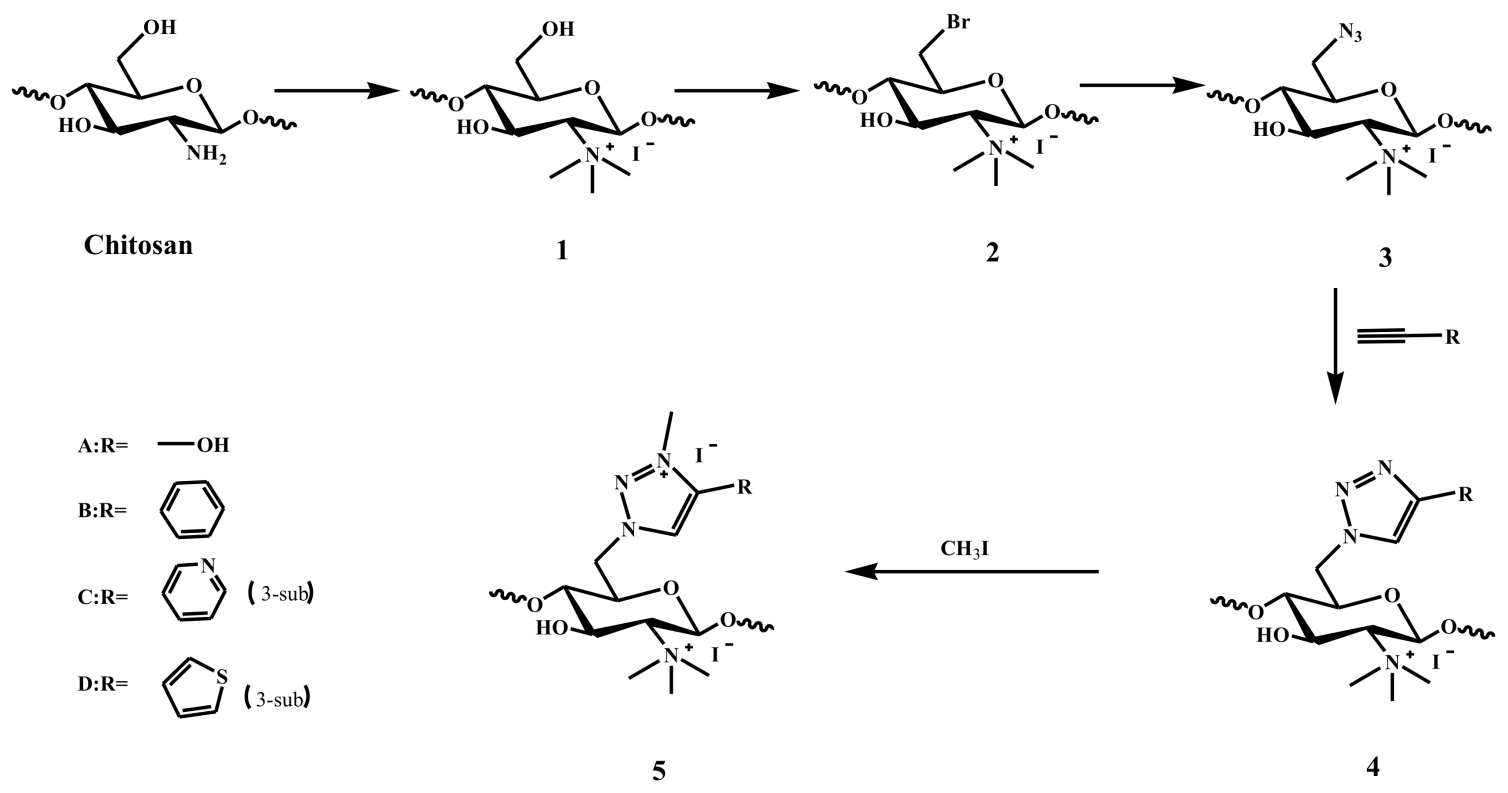
Synthetic routes for the preparation of chitosan derivatives.

**Figure 1:**
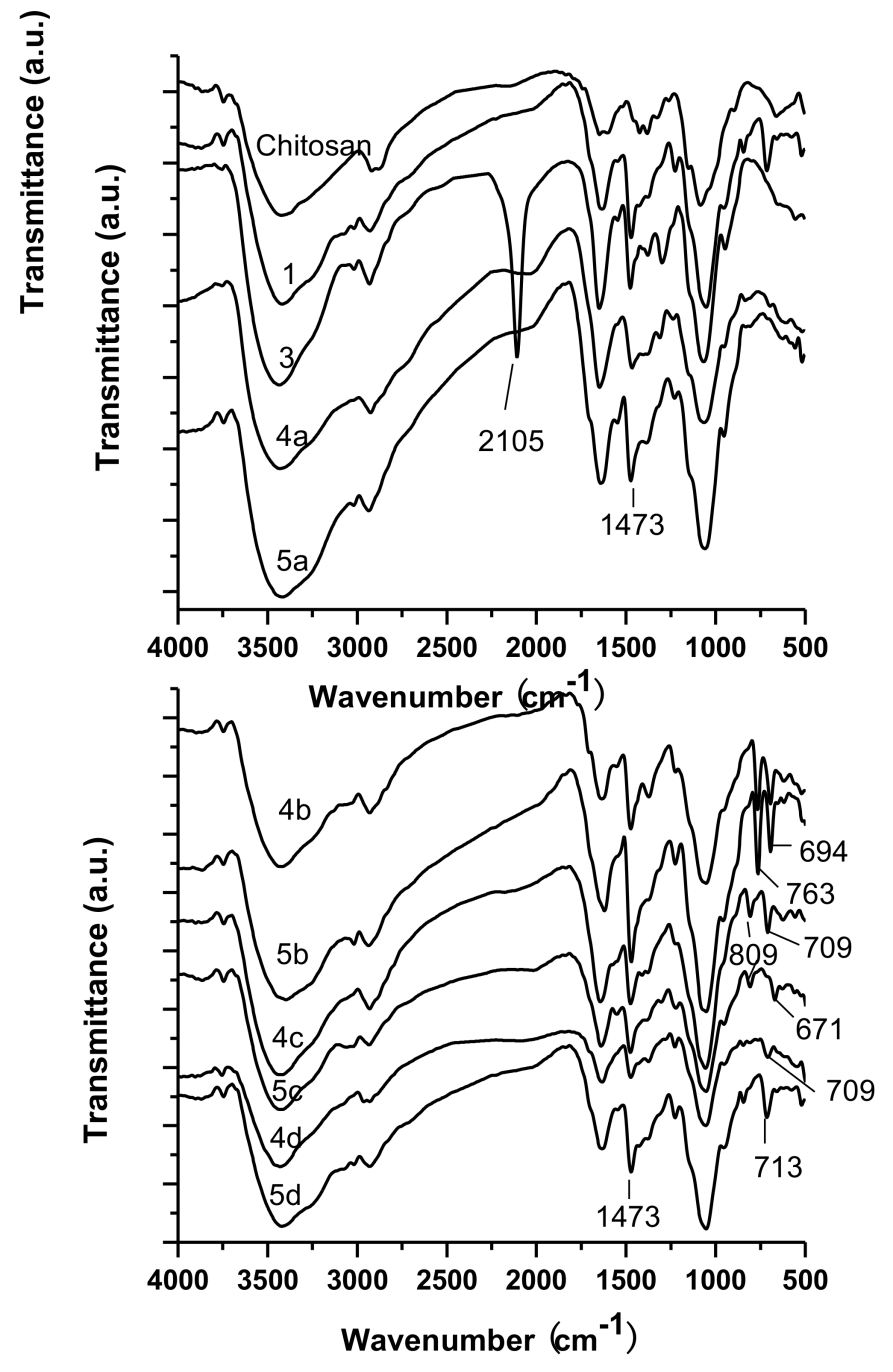
FT-IR spectra of intermediate products and chitosan derivatives **4** (**top**) and **5** (**bottom**).

**Figure 2:**
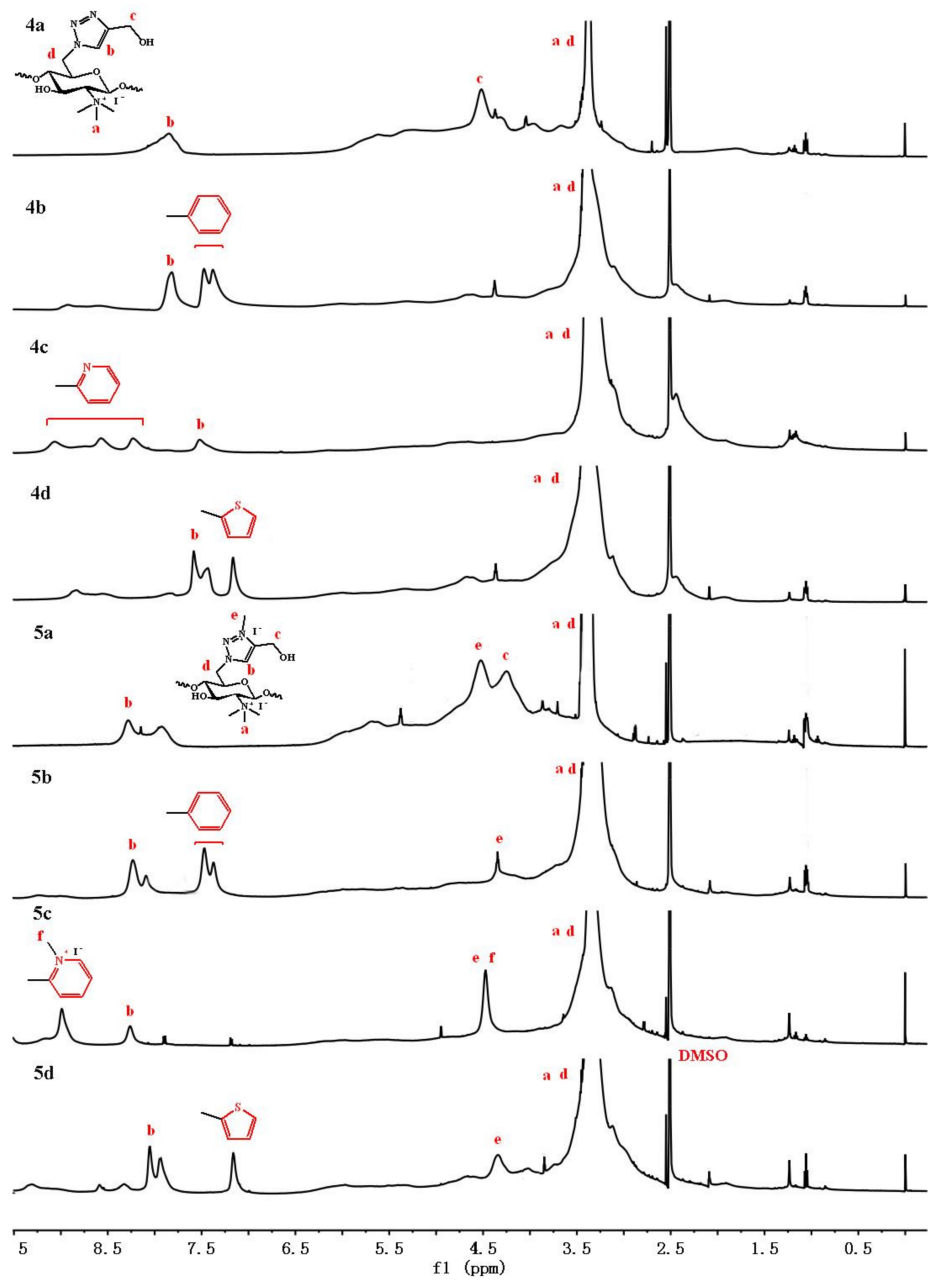
^1^H NMR spectra of chitosan derivatives **4** and **5**.

In the previous work of our group, C_2_-NH_2_ of chitosan was protected by phthaloyl firstly and treated with aqueous hydrazine monohydrate to remove the phthaloyl protecting group after “click reaction” [22,23]. In this way, amino group could be protected well, but more reaction steps would lead to lower overall yield. In this paper, trimethyl quaternary ammonium salt of chitosan was first synthesized through the reaction of the C_2_-NH_2_ of chitosan and iodomethane (Scheme 1). The quaternary ammonium salt was chosen as polymer part by virtue of its water-solubility in neutral and alkaline aqueous solutions. Meanwhile, the quaternization could protect the amino group in the next bromination reaction of C_6_-OH. Each step of synthesis was followed by FT-IR and ^1^H NMR spectroscopy measurements. The FT-IR and ^1^H NMR spectra of compounds **4** and **5** are shown in Figure 1 and Figure 2, respectively.

In the FT-IR spectrum of compound **1** (Figure 1), the peak at 1477 cm^−1^ was ascribed to the deformation vibration of C-H in N-CH_3_ [24]. Then, C-6-Br quaternary ammonium chitosan derivative (**2**) was synthesized by the reaction between the C_6_-OH with NBS and Ph_3_P, as NBS and Ph_3_P could selectively replace primary hydroxyl groups of polysaccharide with bromine [19]. The azidation of compound **2** could be conveniently achieved through a nucleophilic substitution with sodium azide to get 6-azido-6-deoxy-*N*-phthaloyl quaternary ammonium chitosan. The characteristic peak observed at 2105 cm^−1^ was the stretching vibration of -N=N^+^=N^−^ for C-6-azido. As long as we got 6-azido-6-deoxy-*N*-trimethyl quaternary ammonium chitosan, the “click chemistry” could be performed in an elegant way with terminal alkynes with heterocycle as substitutes to synthesize the aimed chitosan derivatives [25]. The peak at 2105 cm^−1^ in spectrum of compound **3** disappeared when the C-6-azido was transformed to 1,2,3-triazoles [26]. New peaks appeared at 767 and 694 cm^−1^ were assigned to the deformation vibration of C-H in benzene in the spectrum of **4b**, new peaks at 806 and 709 cm^−1^ were assigned to the deformation vibration of C-H in pyridine of **4c**, and new peaks at 709 cm^−1^ were assigned to thiophene of **4d**, respectively (Figure 1). In the ^1^H NMR spectra, the appearance of the proton in 1,2,3-triazole at 7.58–7.88 ppm further proved the successful “click reaction” (Figure 2). Meanwhile, the new peaks of heterocycle could be clearly observed at 7.38–7.47 ppm for benzene of **4b**, 8.24–9.07 for pyridine of **4c**, and 7.16 for thiophene of **4d**, respectively. Subsequently, alkylation of 1,2,3-triazolyl chitosan derivative (**4**) was conducted by reacting with iodomethane. In the ^1^H NMR spectra of **5**, the peak corresponding to the proton of the 1,2,3-triazole group shifted from 7.58–7.88 ppm in DMSO-*d*_6_ to 8.18–8.33, and the N-3 methyl protons of the quaternizing group appeared at 4.34–4.55 ppm [27].

### 2.2. Solubility and Radical Scavenging Activity

The application of chitosan is restricted to only acidic conditions where the NH_2_ group becomes protonated [28]. The further enhancement of the bioactivity of chitosan over a broader pH range will promote its application in many areas. The quaternization of chitosan is an important means to improve its solubility. After quaternization, *N*-trimethyl quaternary ammonium chitosan (**2**) showed favorable water solubility. After the alkylation of 1,2,3-triazolyl chitosan derivative (**4**), the water solubility of chitosan derivative (**5**) was further improved. Therefore, compounds **4** and **5** showed better water solubility than chitosan, and could be prepared as aqueous solution (0.01–1.6 mg/mL) at room temperature.

As chemical protectors, antioxidants are classified on the basis of their mode of action as chain breaking or preventive antioxidants. The chain breaking antioxidants are chemical species able to prevent oxidation by acting as free-radical scavengers. In this case, antioxidants directly react with free radicals, producing significantly less reactive species or turning off the radical chain reaction. The preventive antioxidants retard the oxidation process by indirect pathways, including metal chelation, decomposition of hydroperoxides to nonradical species, repairing of primary antioxidants by hydrogen or electron donation, deactivation of singlet oxygen or sequestration of triplet oxygen, and absorption of UV radiation [2].

Here, we tested the radical scavenging activity using different assay systems such as the superoxide radical-scavenging, DPPH radical-scavenging, and hydroxyl radical-scavenging assay. Chitosan has poor solubility in water, so we used water-soluble low molecular chitosan in antioxidant assay.

**Figure 3:**
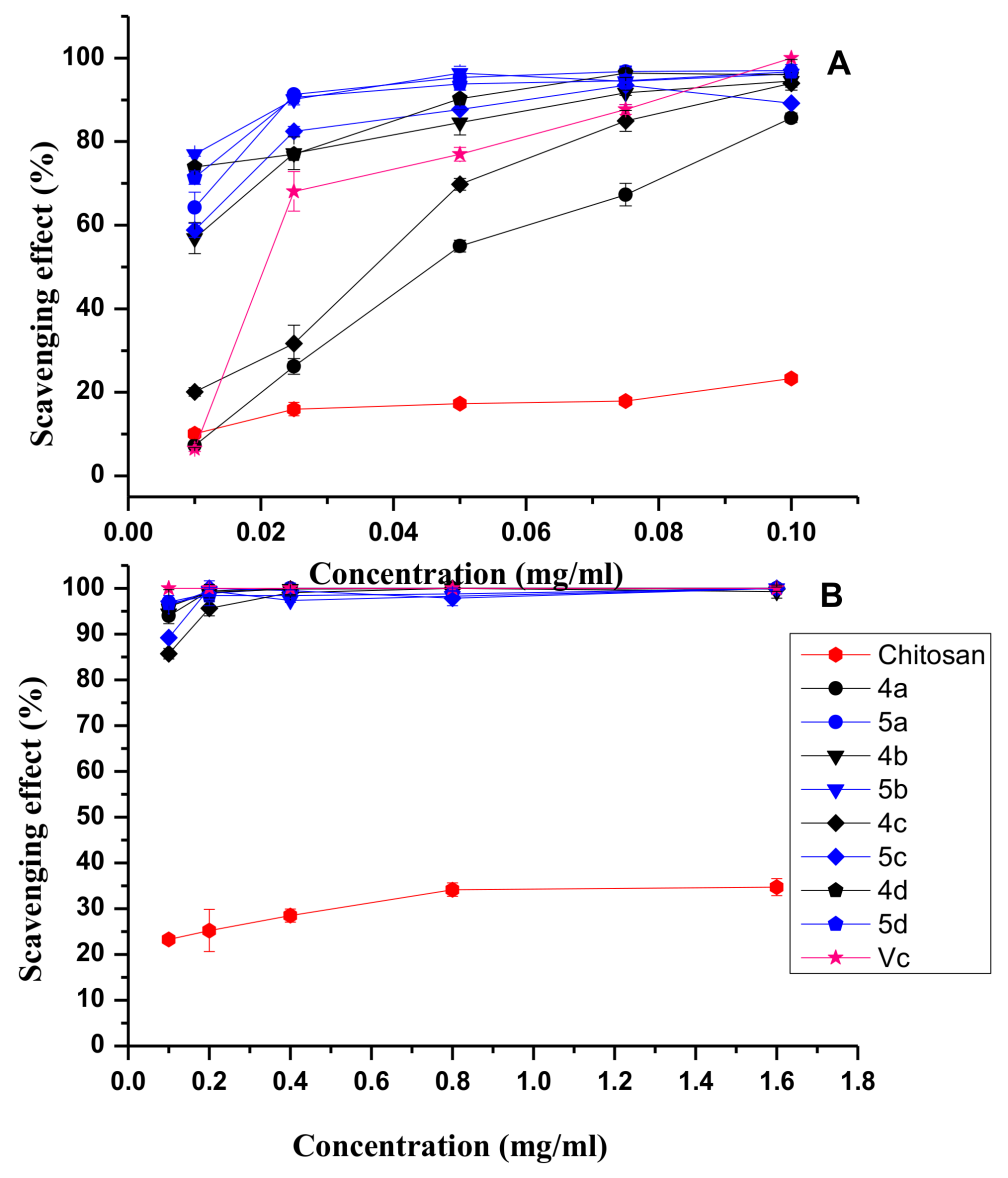
Superoxide-radical scavenging ability of chitosan and chitosan derivatives at different concentrations: (**A**) 0.01–0.1 mg/mL; (**B**) 0.1–1.6 mg/mL.

The superoxide radical scavenging activity of chitosan and its related derivatives was tested by their ability to bleach the superoxide radical generated from the phenazine methosulfate/nicotinamide adenine dinucleotide (PMS/ NADH)reaction (Figure 3) [21]. This assay provides information on the reactivity of test compounds with superoxide free radicals, independently of any enzymatic activity. The generation of superoxide anions was markedly inhibited by Vitamin C with an IC_50_ value of 0.02 mg mL^−1^. Our results clearly demonstrated that the synthesized chitosan derivatives (**4** and **5**) were as effective as Vitamin C in scavenging superoxide radicals. Chitosan showed relatively weak scavenging activity against superoxide radical, and the scavenging index was 34.72% at 1.6 mg mL^−1^. In this test the synthesized chitosan derivatives (**4** and **5**) showed much stronger superoxide radical scavenging ability compared with chitosan. Compound **5** (IC_50_ < 0.01 mg mL^−1^) was more efficient than compound **4** (IC_50_ of **4a** 0.05 mg mL^−1^, IC_50_ of **4c** 0.04 mg mL^−1^, IC_50_ of **4b** and **4d** < 0.01 mg mL^−1^). 

Free radical chain reactions may be inhibited by adding preventive antioxidants that retard the formation of free radicals or stabilize free radicals [21,29]. Owing to the slightly polarized nature of the C(5)-H bond, 1,2,3-triazole has gained recognition as excellent hydrogen donor [30], which can form stable free radicals. Meanwhile, the conjugated double bonds allow electron delocalisation across the molecule thus stabilize the radical [1,31]. Furthermore, it is apparent that the chitosan derivatives with triazolium group (**5**) own better free radical scavenging ability. Because the C(5)-H^…^A^−^ binding ability is strongly enhanced by converting the triazole unit into a triazolium cation, the latter is expected to be a more efficient anion captor [21], which may help stabilize free radicals. The possible action mechanisms may be hydrogen-atom transfer and radical adduct formation [2].

**Figure 4:**
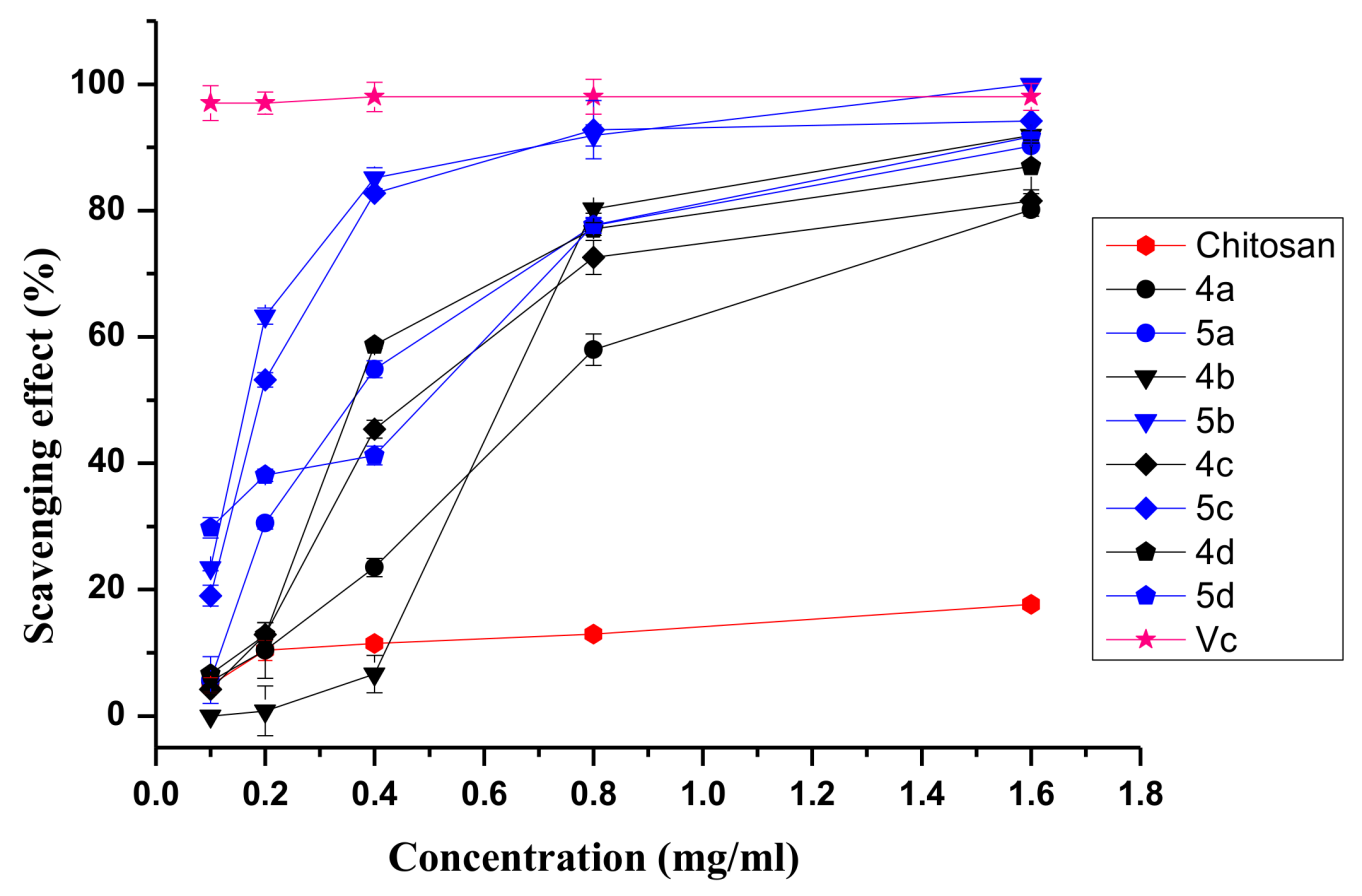
1,1-diphenyl-2-picrylhydrazyl (DPPH)-radical scavenging ability of chitosan and chitosan derivatives.

The DPPH radical scavenging activity of chitosan and derivatives synthesized was also evaluated based on their ability to bleach the stable radical DPPH (Figure 4). This assay provided information on the reactivity of the compounds with a stable free radical. Because of the odd electron, DPPH shows a strong absorption band at 517 nm in visible spectroscopy. As this electron becomes paired off in the presence of a free radical scavenger, the absorption vanishes, and the resulting decolorization is stoichiometric with respect to the number of electrons taken up [1,31]. As a positive control, Vitamin C was tested with IC_50_ < 0.1 mg mL^−1^. Test results showed that chitosan, compound **4**, and compound **5** inhibited DPPH anion formation in a concentration dependent manner, but compound **5** showed more potent scavenging activity (IC_50_ 0.17–0.51 mg mL^−1^), followed by compound **4** (IC_50_ 0.36–0.72 mg mL^−1^), and chitosan (17.67% at 1.6 mg mL^−1^), respectively. Recently, chemical modification of polysaccharides is increasingly reported for its potential of improving the biological activity of polysaccharides. The experimental data above and related literatures demonstrated that the chemical modification of polysaccharides was conducive to improving the free radical scavenging activity of them. 

**Figure 5:**
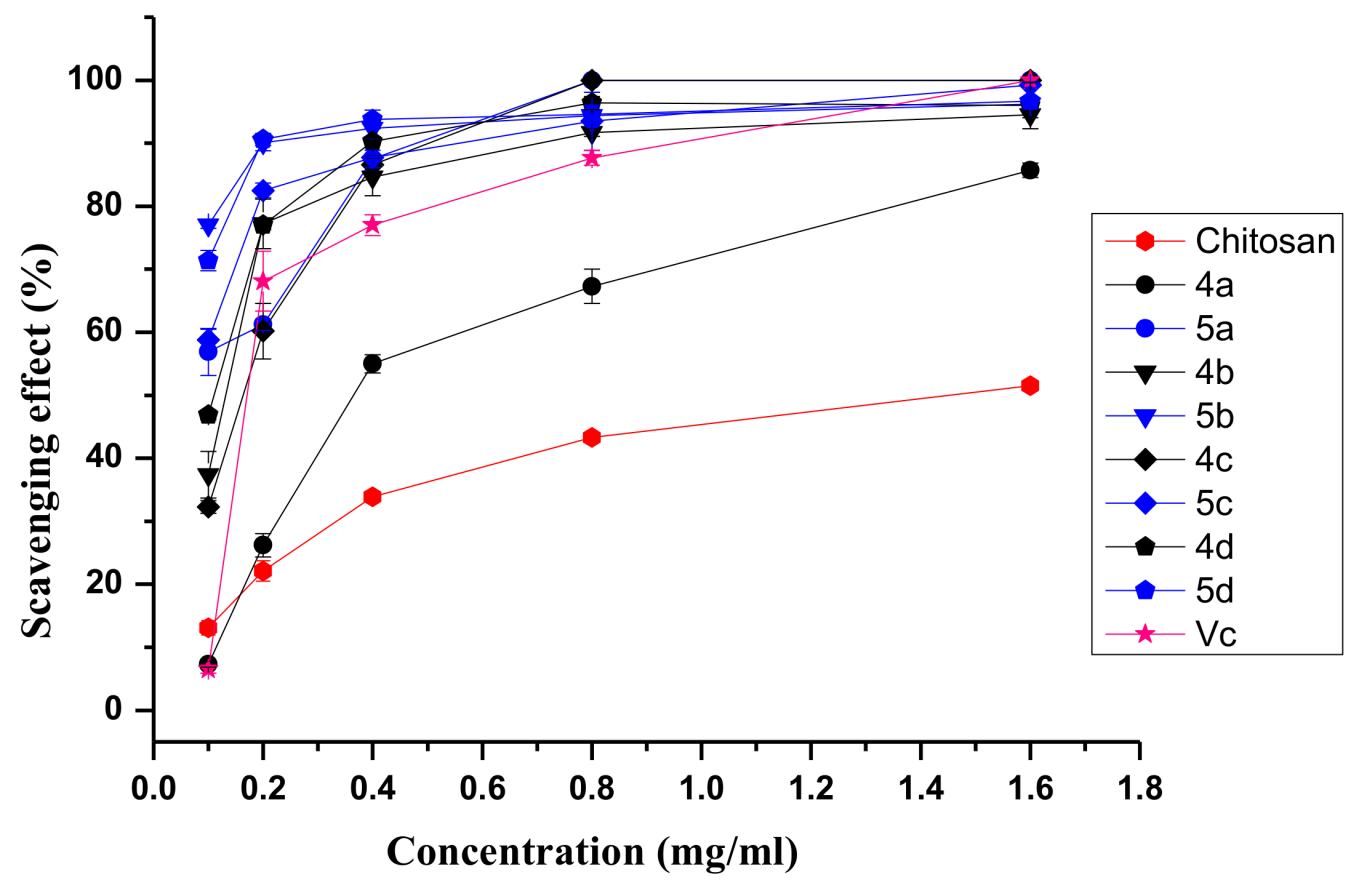
Hydroxyl-radical scavenging ability of chitosan and chitosan derivatives.

Among the oxygen-centered radicals, hydroxyl radical is the most electrophilic and reactive. It is a highly potent oxidant that can react with almost all biomolecules found in living cells. Figure 5 shows the hydroxyl radical scavenging ability of chitosan and the synthesized derivatives at various concentrations. The synthesized chitosan derivatives (**4** and **5**) also showed much stronger hydroxyl radical scavenging ability compared with chitosan in a concentration-dependent manner. The inhibitory activity was observed in the following order: compound **5** (IC_50_ < 0.1 mg mL^−1^) > compound **4** (IC_50_ 0.11–0.36 mg mL^−1^) > chitosan (IC_50_ 1.53 mg mL^−1^). The results further confirmed that triazole or triazolium groups grafted into the synthesized chitosan derivatives contributed a lot to the radical scavenging action and consequently increased the radical scavenging activity. Superoxide indirectly initiates lipid peroxidation because superoxide anion acts as a precursor of singlet oxygen and hydroxyl radical. Hydroxyl radicals eliminate hydrogen atoms from the membrane lipid, which results in lipid peroxidation. Based on its better free radical-scavenging activity in our experiments, compound **5** would have been expected to be superior to compound **4** in lipid peroxidation and the protective effect on oxidative damage induced by H_2_O_2_ in cells.

On the other hand, the inconsistent relative radical scavenging activity of the synthesized chitosan derivatives against different free radical may be related to the different reaction mechanisms in different systems. There are some fundamental differences among the three assays. First, the features of the oxidant such as their redox potentials or stability are not the same. The scavenging effect on DPPH radicals and superoxide radicals represent direct radical scavenging activity. In the hydroxyl radical scavenging assay, hydroxyl radicals are generated by the Fenton reaction and the inhibition could be attributed to the inhibition of radicals or the Fe^2+^ chelating effectof the test compounds. Second, other factors such as the surface activity affected by the polymer structures and the different reaction mechanisms in different systems may also affect the ability of test compounds to react with and quench different radicals [1].

## 3. Materials and Methods

### 3.1. Materials

Chitosan was purchased from Qingdao Baicheng Biochemical Corp. (Qingdao, China). Its degree of deacetylation is 90% and the viscosity-average molecular weight is 7.0 × 10^4^ D. Terminal alkynes (propargyl alcohol, ethynylbenzene, 3-ethynylpyridine, and 3-ethynylthiophene) were purchased from Merck Life Science (Shanghai) Co., Ltd., (Shanghai, China) with a minimum purity of 98%. The other reagents such as iodomethane, sodium iodide, sodium hydroxide, cuprous iodide, potassium iodide, potassium bromide (KBr), and solvents are analytical grade and were supplied by Sinopharm Chemical Reagent Co., Ltd., Shanghai, China.

### 3.2. Analytical Methods

FT-IR spectra were measured on a Jasco-4100 Fourier Transform Infrared Spectroscopy (Tokyo, Japan), provided by JASCO Co., Ltd., Shanghai, China) with KBr disks. ^1^H Nuclear Magnetic Resonance (^1^H NMR) was measured with a Bruker AVIII-500 Spectroscopy with TCI Cryo Probe (Bruker Tech. and Serv. Co., Ltd., Beijing, China). The elemental analyses (C, H, and N) were performed on a Vario Micro Elemental Analyzer (Elementar, Langenselbold, Germany). The Degree of Substitution (DS) was calculated based on elemental analysis results.

### 3.3. The synthesis of Chitosam Derivatives

#### 3.3.1. Synthesis of 6-Azido-6-deoxy-*N*-trimethyl Quaternary Ammonium Chitosan (**3**)

The synthetic routes for the preparation of chitosan derivatives are shown in Scheme 1.

*N*-trimethyl quaternary ammonium chitosan (Compound **1**): chitosan (1.8 g, 12 mmol), 5.4 g *N*-bromobutanimide, 8.55 g triphenylphosphine 16 mL of aqueous sodium hydroxide solution (15%, *w*/*v*), and 18 mL of iodomethane were added to 100 mL of *N*-methyl-2-pyrrolidone (NMP) and stirred at 60 °C under an argon atmosphere for 2 h. The mixture was precipitated into ethanol, and the precipitate was collected by filtration and washed by ethanol. The products were dried at 60 °C for 24 h, yield: 71.1%; FT-IR (KBr film): *v* 3421 (NH_2_ and OH), *v* 1469 (C-H of quaternary ammonium). ^1^H NMR (500 MHz, DMSO-*d*_6_): δ 3.27 ppm (C**H**_3_-N); δ 3.34–5.26 (pyranose rings).

6-Bromo-6-deoxy-*N*-trimethyl quaternary ammonium chitosan (Compound **2**): compound **1** (2.08 g, 6.5 mmol), 5.78 g sodium iodide, 12 mL of aqueous sodium hydroxide solution (15%, *w*/*v*), and 18 mL of iodomethane were added to 100 mL of NMP and stirred at 60 °C under an argon atmosphere for 2 h. The mixture was precipitated into ethanol, and the precipitate was collected by filtration. The unreacted NBS, TPP, and other outgrowth were extracted in a Soxhlet apparatus with ethanol and acetone for 48 h, respectively. The products were dried at 60 °C for 24 h, yield: 67.9%; ^1^H NMR (500 MHz, DMSO-*d*_6_): δ 3.44 ppm (C**H**_3_-N); δ 3.34–5.26 (pyranose rings), 3.75 (C**H**_2_Br) ppm.

6-Azido-6-deoxy*-N*-trimethyl quaternary ammonium chitosan (Compound **3**): compound **2** (0.788 g, 2 mmol), and 0.65 g sodium azide were added to 45 mL of NMP and stirred for 4 h at 80 °C under an argon atmosphere. The mixture was precipitated into ethanol, and the precipitate was collected by filtration. After being extracted in a Soxhlet apparatus with ethanol for 48 h and being dialyzed against deionized water for 48 h to remove the probable remained sodium azide, the products were dried at 60 °C for 24 h, yield: 67.9%; FT-IR (thin film): *v* 3432 (NH_2_ and OH), *v* 2105 (C-6-azido), *v* 1477 (C-H of quaternary ammonium); ^1^H NMR (500 MHz, DMSO-*d*_6_): δ 3.44 ppm (C**H**_3_-N); δ 3.34–5.26 (pyranose rings), 4.0 (C**H**_2_N_3_) ppm.

#### 3.3.2. Synthesis of Chitosan Derivative Bearing 1,2,3-Triazole (**4**) and 1,2,3-Triazolium (**5**)

Compound **4**: compound 3 (1.0 mmol), 12 mg cuprous iodide, terminal alkynes (propargyl alcohol, ethynylbenzene, 3-ethynylpyridine, and 3-ethynylthiophene) (2.0 mmol), and 3 mL of triethylamine were added to 20 mL of dimethyl sulfoxide and stirred at 75 °C under an argon atmosphere for 48 h. The mixture was filtered and the filtrate was collected and precipitated into ethanol. The precipitate was washed with saturated solution of potassium iodide and filtered. The unreacted alkyne was extracted in a Soxhlet extractor with ethanol for 48 h. The products were dried at 60 °C for 24 h. 

**4a**: yield: 72.6%; DS: 0.81; FT-IR (thin film): *v* 3432 (NH_2_ and OH), *v* 1465 (C-H of quaternary ammonium); ^1^H NMR (500 MHz, DMSO-*d*_6_): δ 3.4 ppm (C**H**_3_-N^+^); δ 4.53 (triazole-C**H**_2_-O**H**); δ 7.88 (triazole-5-**H**).

**4b**: yield: 51.9%; DS: 0.75; FT-IR (thin film): *v* 3424 (NH_2_ and OH), *v* 1473 (C-H of quaternary ammonium), *v* 767 and 694 (C-H of benzene); ^1^H NMR (500 MHz, DMSO-*d*_6_): δ 3.4 ppm (C**H**_3_-N^+^); δ 7.38–7.47 (benzene-**H**); 7.82 (triazole-5-**H**).

**4c**: yield: 51.9%; DS: 0.72; FT-IR (thin film): *v* 3421 (NH_2_ and OH), *v* 1473 (C-H of quaternary ammonium), *v* 806 and 709 (C-H of pyridine); ^1^H NMR (500 MHz, DMSO-*d*_6_): δ 3.4 ppm (C**H**_3_-N^+^); 7.53 (triazole-5-**H**); δ 8.24–9.07 (pyridine-**H**).

**4d**: yield: 51.9%; DS: 0.83; FT-IR (thin film): *v* 3428 (NH_2_ and OH), *v* 1473 (C-H of quaternary ammonium), *v* 709 (C-H of thiophene); ^1^H NMR (500 MHz, DMSO-*d*_6_): δ 3.4 ppm (C**H**_3_-N^+^); δ 7.16 (thiophene-**H**); 7.58 (triazole-5-**H**).

Chitosan derivative bearing 1,2,3-triazolium (Compound **5**) were prepared according to the methods reported by Tan [32]. A solution of compound **4** (1 mmol) and iodomethane (0.187 mL, 3 mmol) in 15 mL of DMSO was stirred at 60 °C for 24 h. Afterwards, the remaining iodomethane was evaporated, and the reaction mixture was precipitated into 100 mL of acetone. The solid product was filtered, washed with acetone three times. After being dialyzed against deionized water for 48 h, the chitosan derivative (**5**) was obtained by lyophilization of their aqueous solutions. 

**5a**: yield: 98.5%; DS: 0.72; FT-IR (thin film): *v* 3417 (NH_2_ and OH), *v* 1473 (C-H of quaternary ammonium); ^1^H NMR (500 MHz, DMSO-*d*_6_): δ 3.4 ppm (C**H**_3_-N^+^); δ 4.25 (triazole-C**H**_2_-O**H**); δ 4.55 (triazolium-C**H**_3_); δ 8.31 (triazole-5-**H**).

**5b**: yield: 94.1%; DS: 0.72; FT-IR (thin film): *v* 3397 (NH_2_ and OH), *v* 1469 (C-H of quaternary ammonium), *v* 763 and 694 (C-H of benzene); ^1^H NMR (500 MHz, DMSO-*d*_6_): δ 3.4 ppm (C**H**_3_-N^+^); δ 4.34 (triazolium-C**H**_3_); δ 7.37–7.47 (benzene-**H**); δ 8.33 (triazole-5-**H**). 

**5c**: yield: 73.5%; DS: 0.69; FT-IR (thin film): *v* 3421 (NH_2_ and OH), *v* 1477 (C-H of quaternary ammonium), *v* 809 and 671 (C-H of pyridine); ^1^H NMR (500 MHz, DMSO-*d*_6_): δ 3.4 ppm (C**H**_3_-N^+^); δ 4.47 (triazolium and pyridinium-C**H**_3_); δ 8.99 (pyridine-**H**); δ 8.27 (triazole-5-**H**). 

**5d**: yield: 73.5%; DS: 0.75; FT-IR (thin film): 3421 (NH_2_ and OH), *v* 1473 (C-H of quaternary ammonium), *v* 713 (C-H of thiophene); ^1^H NMR (500 MHz, DMSO-*d*_6_): δ 3.4 ppm (C**H**_3_-N^+^); δ 4.41 (triazolium-C**H**_3_); δ 7.16 (thiophene-**H**); 8.18 (triazole-5-**H**).

### 3.4. Antioxidant Assay

#### 3.4.1. DPPH-Radical Scavenging Ability Assay

The DPPH-radical scavenging capacity of the products were evaluated by the following method [33]: DPPH in ethanol (180 μmol/L) and sample solution (10 mg/ mL) were first prepared. The reaction mixture, a total volume of 3.0 mL, containing the samples solution (0.03, 0.06, 0.12, 0.24 and 0.48 mL), were incubated with water (0.97, 0.94, 0.88, 0.76 and 0.52 mL), and DPPH (2 mL) at 25 °C for 30 min. The concentration of hydroxyl-radical was 0.1, 0.2, 0.4, 0.8, and 1.6 mg/ml, respectively. Then, the absorbance of the remained DPPH radical was measured at 517 nm against a blank. Three replicates for each sample concentration were tested and the scavenging effect was obtained according to the following equation:

$$\mathrm{Scavenging}\;\mathrm{effect}\;\left(\text{\%}\right)=\left[1-\frac{A_{\mathrm{sample}\;517\mathrm{nm}}-A_{\mathrm{control}\;517\mathrm{nm}}}{A_{\mathrm{blank}\;517\mathrm{nm}}}\right]\times 100\text{\%}$$

where A_sample 517nm_ is the absorbance of the sample at 517 nm, A_blank 517nm_ is the absorbance of the blank at 517 nm and A_control 517nm_ represents the absorbance of the control (distilled water instead of DPPH) at 517 nm. The antioxidant activity was expressed as IC_50_, which was defined as the concentration of compound required for inhibition of the radical formation by 50%. Vitamin C was used as the positive control. 

#### 3.4.2. Superoxide-Radical Scavenging Ability Assay

The superoxide radical scavenging ability was assessed following Xing’s methods with minor modification [34]. The Tris-HCl buffer (16 mM, pH 8.0) and sample solution (1 mg/mL and 5 mg/mL) were first prepared. Then the solution of nicotinamide adenine dinucleotide reduced (NADH 365.7 μg/mL), nitro blue tetrazolium (NBT 245.3 μg/mL), and phenazine mothosulfate (PMS 18.38 μg/mL) were prepared in Tris-HCl buffer (pH = 8.0). The reaction mixture, a total volume 3.0 mL, containing the samples solution (A: 1 mg/ mL, 0.03, 0.075, 0.15, and 0.225 mL; B: 5 mg/ mL, 0.06, 0.12, 0.24, 0.48 and 0.96 mL), were incubated with Tris-HCl buffer (A: 1.47, 1.425, 1.35 and 1.225; B: 1.44, 1.38, 1.26, 1.02 and 0.54 mL), NADH (0.5 mL), NBT (0.5 mL), and PMS (0.5 mL) at 25 °C for 5 min. The concentration of hydroxyl-radical was 0.01, 0.025, 0.05, 0.075, 0.1, 0.2, 0.4, 0.8, 1.6 mg/mL, respectively. The absorbance was read at 560 nm against blank. Three replicates for each sample were tested and the capability of scavenging superoxide radical was calculated using the following equation:

$$\mathrm{Scavenging}\;\mathrm{effect}\;\left(\text{\%}\right)=\left[1-\frac{A_{\mathrm{sample}\;560\mathrm{nm}}-A_{\mathrm{control}\;560\mathrm{nm}}}{A_{\mathrm{blank}\;560\mathrm{nm}}}\right]\times 100\text{\%}$$

where A_sample 517nm_ is the absorbance of the sample at 560 nm, and A_control 560nm_ is the absorbance of the negative control (distilled water instead of NADH for each concentration) and A_blank 560nm_ is the absorbance of the blank (distilled water instead of the samples). The superoxide radical-scavenging activity was expressed as the IC_50_ value. Vitamin C was used as a positive control.

#### 3.4.3. Hydroxyl-Radical Scavenging Ability Assay

The test of hydroxyl-radical scavenging ability was carried out according to Liu’s methods with minor modification [35]. The phosphate-buffered saline (pH = 7.4) and sample solution (10 mg/ mL) were first prepared. Then the solution of H_2_O_2_ (3%) and safranine T (360 μg/mL) were prepared in phosphate-buffered saline (pH = 7.4). The solution of EDTA-Fe^2+^ (2 mmol/L) was prepared in water. The reaction mixture, a total volume 4.5 mL, containing sample solution (0.045, 0.09, 0.18, 0.36 and 0.72 mL), were incubated with water (0.955, 0.91, 0.82, 0.64 and 0.28 mL), EDTA-Fe^2+^ solution (0.5 mL), safranine T (1 mL), and H_2_O_2_ (1 mL) in potassium phosphate buffer (0.51 mL, pH 7.4) at 37 °C for 30 min. The concentration of hydroxyl-radical was 0.1, 0.2, 0.4, 0.8, 1.6 mg/ml, respectively. The absorbance of the mixture was measured at 520 nm. In the blank, samples were substituted with distilled water. Meanwhile, in the negative control, H_2_O_2_ was substituted with potassium phosphate buffer. Three replicates for each sample were tested. The capability of scavenging hydroxyl radicals of the products was computed using the following equation:

$$\mathrm{Scavenging}\;\mathrm{effect}\left(\text{\%}\right)=\frac{A_{\mathrm{sample}\;520\mathrm{nm}}-A_{\mathrm{blank}\;520\mathrm{nm}}}{A_{\mathrm{control}\;520\mathrm{nm}}-A_{\mathrm{blank}\;520\mathrm{nm}}}\times 100\text{\%}$$

where A_blank 520nm_ is the absorbance of the blank at 520 nm; A_sample 520nm_ is the absorbance of the sample at 520 nm; A_control 520nm_ is the absorbance of the control at 520 nm. The antioxidant activity of test compounds was expressed as IC_50_. Vitamin C was used as a positive control.

Each experiment was performed in three replicates and the data were expressed as mean ± standard deviation (SD). Significant difference analysis was performed using Duncan’s multiple range test. A level of *p* < 0.05 was considered as statistically significant.

## 4. Conclusions

We investigated the possible radical scavenging ability of chitosan and its derivatives with 1,2,3-triazole or 1,2,3-triazolium because these groups may improve the antioxidant property of chitosan. Firstly, we designed and synthesized a group of novel water soluble chitosan derivatives containing 1,2,3-triazole or 1,2,3-triazolium. Through chemical modification, chitosan was derivatized with hydrophilic group (quaternary ammonium salt) and biologically active group (triazole or triazolium), which enabled the product to have better antioxidant property and water solubility. The radical scavenging activity against three kinds of free radicals was tested. All the chitosan derivatives exhibited higher radical scavenging activity than chitosan. Moreover, the triazolium group was found to be a more efficient group than triazole and contributed a lot to the radical scavenging ability of chitosan derivatives. The experiment data demonstrated that the chemical modification of chitosan with triazolium functional groups was conducive to improving the antioxidant activity of chitosan. These findings mentioned above bring further evidence that chitosan derivatives are active and have the potential of becoming alternatives of free radical scavenger.

## Abbreviations

The following abbreviations are used in this manuscript:

DPPH	1,1-Diphenyl-2-picrylhydrazyl
DMSO	Dimethyl Sulphoxide
DMF	*N*,*N*-Dimethylformamide
EDTA	Ethylenediaminetetraacetic acid
NMP	*N*-Methyl pyrrolidone
